# Supplementary material for: Single Cell Transcriptomic and Chromatin Profiles Suggest Layer Vb Is the Only Layer With Shared Excitatory Cell Types in the Medial and Lateral Entorhinal Cortex
Source: Front Neural Circuits. 2022 Jan 26;15:806154. doi: 10.3389/fncir.2021.806154 (PMC8826650; doi:10.3389/fncir.2021.806154)
Supplement: Supplementary file 14 [file Data_Sheet_1.ZIP › MEC_Sup_vs_LEC_Sup_Motifs/homerResults.html]

MEC\_Sup\_vs\_LEC\_Sup\_Motifs// - Homer de novo Motif Results


# Homer *de novo* Motif Results (MEC\_Sup\_vs\_LEC\_Sup\_Motifs//)

Known Motif Enrichment Results  
Gene Ontology Enrichment Results  
If Homer is having trouble matching a motif to a known motif, try copy/pasting the matrix file into
STAMP  
More information on motif finding results: HOMER
| Description of Results
| Tips
  
Total target sequences = 21073  
Total background sequences = 12205  
\* - possible false positive  

|  |  |  |  |  |  |  |  |  |
| --- | --- | --- | --- | --- | --- | --- | --- | --- |
| Rank | Motif | P-value | log P-pvalue | % of Targets | % of Background | STD(Bg STD) | Best Match/Details | Motif File |
| 1 | G C T A T A C G A G T C C G T A A C T G A C T G A C G T A C T G A G C T C T G A | 1e-2301 | -5.299e+03 | 53.11% | 20.85% | 222.3bp (263.8bp) | E2A(bHLH),near\_PU.1/Bcell-PU.1-ChIP-Seq(GSE21512)/Homer(0.982) More Information \| Similar Motifs Found | motif file (matrix) |
| 2 | A G C T A G C T G T A C A T G C T C G A C A G T G T C A A G C T A T C G A C T G A G C T G A C T | 1e-142 | -3.292e+02 | 21.79% | 15.16% | 229.6bp (251.1bp) | TCF21(var.2)/MA1568.1/Jaspar(0.869) More Information \| Similar Motifs Found | motif file (matrix) |
| 3 | C A G T A C T G C T A G C G T A T G C A C T G A C A G T A C T G A G C T T G A C | 1e-136 | -3.140e+02 | 35.10% | 27.26% | 258.9bp (258.1bp) | NR4A1/MA1112.2/Jaspar(0.823) More Information \| Similar Motifs Found | motif file (matrix) |
| 4 | C G T A C G T A C G T A A G C T G T C A A C G T A C T G A C T G | 1e-76 | -1.756e+02 | 33.99% | 28.14% | 247.1bp (257.7bp) | Twist2/MA0633.1/Jaspar(0.718) More Information \| Similar Motifs Found | motif file (matrix) |
| 5 | G C A T C G T A A G T C G T C A A G C T T G C A A G C T C G T A A G C T T C G A | 1e-75 | -1.740e+02 | 21.23% | 16.36% | 261.5bp (232.4bp) | PB0080.1\_Tbp\_1/Jaspar(0.684) More Information \| Similar Motifs Found | motif file (matrix) |
| 6 | T G A C C G A T A C T G A T C G T C G A A G T C C G A T C A T G A C T G C T G A A G C T G T A C | 1e-70 | -1.625e+02 | 0.36% | 0.02% | 183.6bp (113.3bp) | Hand2(bHLH)/Mesoderm-Hand2-ChIP-Seq(GSE61475)/Homer(0.631) More Information \| Similar Motifs Found | motif file (matrix) |
| 7 | C G T A T A G C C T A G G C A T T G C A C G T A G T C A C T G A | 1e-66 | -1.525e+02 | 24.49% | 19.64% | 249.0bp (240.7bp) | GMEB2/MA0862.1/Jaspar(0.772) More Information \| Similar Motifs Found | motif file (matrix) |
| 8 | C A G T C G T A A G T C A G T C A C G T A C T G C A T G G A T C A C G T C A T G A C G T G A C T | 1e-57 | -1.335e+02 | 0.39% | 0.04% | 186.1bp (60.3bp) | ZEB1(Zf)/PDAC-ZEB1-ChIP-Seq(GSE64557)/Homer(0.698) More Information \| Similar Motifs Found | motif file (matrix) |
| 9 | A C T G C G T A G T C A A G T C A T G C A G T C C T G A A T C G G T C A C G A T A T C G A G T C | 1e-55 | -1.282e+02 | 0.42% | 0.04% | 160.8bp (138.0bp) | VDR/MA0693.2/Jaspar(0.653) More Information \| Similar Motifs Found | motif file (matrix) |
| 10 | T A C G G T A C C G A T C T A G A C T G G A T C G A T C C T G A | 1e-55 | -1.270e+02 | 17.84% | 13.96% | 259.5bp (272.2bp) | Bcl11a(Zf)/HSPC-BCL11A-ChIP-Seq(GSE104676)/Homer(0.738) More Information \| Similar Motifs Found | motif file (matrix) |
| 11 | C G T A A T G C G C A T C A G T T C G A T A G C G A T C C G A T C G A T G T A C | 1e-54 | -1.261e+02 | 15.42% | 11.80% | 255.9bp (262.4bp) | SD0001.1\_at\_AC\_acceptor/Jaspar(0.876) More Information \| Similar Motifs Found | motif file (matrix) |
| 12 | C G T A A C G T A C T G A G T C C T G A C T G A C G T A A C G T | 1e-50 | -1.171e+02 | 9.95% | 7.14% | 244.1bp (259.2bp) | POU2F2/MA0507.1/Jaspar(0.959) More Information \| Similar Motifs Found | motif file (matrix) |
| 13 | A T G C C G A T G A T C A G C T G T C A C A G T T C A G G C A T A C G T G T A C G T C A A C G T | 1e-50 | -1.166e+02 | 0.39% | 0.04% | 227.0bp (104.6bp) | ZNF768(Zf)/Rajj-ZNF768-ChIP-Seq(GSE111879)/Homer(0.655) More Information \| Similar Motifs Found | motif file (matrix) |
| 14 | T C A G C G T A C A G T C T A G A C T G A C T G T G A C G C A T G A C T G T C A A T G C G A T C | 1e-43 | -9.975e+01 | 0.33% | 0.04% | 269.1bp (53.2bp) | ZNF449/MA1656.1/Jaspar(0.689) More Information \| Similar Motifs Found | motif file (matrix) |
| 15 | C G T A A G T C G A T C A C G T A G T C C G A T A G T C A G T C A C G T A T G C A C G T A C T G | 1e-40 | -9.265e+01 | 0.28% | 0.03% | 271.2bp (95.0bp) | PPARa(NR),DR1/Liver-Ppara-ChIP-Seq(GSE47954)/Homer(0.606) More Information \| Similar Motifs Found | motif file (matrix) |
| 16 | T A G C A T G C C T A G G A C T C G T A G T C A A C G T T A C G A C G T G C A T | 1e-37 | -8.539e+01 | 0.27% | 0.03% | 223.1bp (149.6bp) | PB0141.1\_Isgf3g\_2/Jaspar(0.603) More Information \| Similar Motifs Found | motif file (matrix) |
| 17 | G C T A T G A C G T A C G T C A T A G C T C A G C G T A T G A C G A T C G T A C G T A C G T C A | 1e-36 | -8.462e+01 | 0.23% | 0.02% | 238.9bp (106.3bp) | KLF17/MA1514.1/Jaspar(0.713) More Information \| Similar Motifs Found | motif file (matrix) |
| 18 | G T C A A C G T A G T C A G T C G T C A A C T G A G T C C G T A C G T A C G A T C G T A A G T C | 1e-35 | -8.064e+01 | 0.26% | 0.03% | 153.7bp (172.3bp) | ZNF140/MA1589.1/Jaspar(0.635) More Information \| Similar Motifs Found | motif file (matrix) |
| 19 | G T C A T C G A A C G T T C A G G C T A G A T C C T A G C T G A A C T G T G A C G T A C G T C A | 1e-34 | -7.954e+01 | 0.28% | 0.04% | 246.8bp (253.9bp) | PB0108.1\_Atf1\_2/Jaspar(0.628) More Information \| Similar Motifs Found | motif file (matrix) |
| 20 | T C A G G C A T C G T A C A T G T A C G T G A C G T A C G C T A A T G C C A T G A T G C G A T C | 1e-34 | -7.937e+01 | 0.22% | 0.02% | 205.8bp (62.3bp) | Klf12/MA0742.1/Jaspar(0.737) More Information \| Similar Motifs Found | motif file (matrix) |
| 21 | C G A T A T C G G T A C C A T G C A T G G T A C G A T C G T A C C G A T A T C G T C G A C A G T | 1e-33 | -7.777e+01 | 0.17% | 0.01% | 142.3bp (99.2bp) | THAP1/MA0597.1/Jaspar(0.627) More Information \| Similar Motifs Found | motif file (matrix) |
| 22 | A G T C G T A C G T C A C G T A A T G C A G T C C G A T C G A T A G T C G T C A A G C T T C G A | 1e-31 | -7.365e+01 | 0.24% | 0.03% | 263.6bp (89.1bp) | PB0029.1\_Hic1\_1/Jaspar(0.626) More Information \| Similar Motifs Found | motif file (matrix) |
| 23 | A G T C A C G T C G T A C G T A A C G T A C G T A G T C A G T C C G T A A G T C | 1e-29 | -6.698e+01 | 0.35% | 0.07% | 255.9bp (178.0bp) | RELB/MA1117.1/Jaspar(0.688) More Information \| Similar Motifs Found | motif file (matrix) |
| 24 | A C T G A C G T A T G C A G T C A T C G A C T G A C G T T A G C A G T C A C G T | 1e-29 | -6.684e+01 | 0.23% | 0.03% | 236.0bp (36.4bp) | ETS2/MA1484.1/Jaspar(0.629) More Information \| Similar Motifs Found | motif file (matrix) |
| 25 | C G T A C T A G C G A T G C T A G A T C G C A T G C T A G A C T G C T A A C G T G A T C G A T C | 1e-28 | -6.578e+01 | 0.15% | 0.01% | 175.2bp (6.1bp) | PRDM1/MA0508.3/Jaspar(0.623) More Information \| Similar Motifs Found | motif file (matrix) |
| 26 | C A T G A T C G G T C A C G A T C A G T C A T G A C T G C A G T G A T C G T C A T G C A C A G T | 1e-27 | -6.285e+01 | 0.15% | 0.01% | 249.1bp (157.2bp) | NFYA/MA0060.3/Jaspar(0.691) More Information \| Similar Motifs Found | motif file (matrix) |
| 27 | A C G T A C T G C G T A A C T G A C T G C T A G C G T A A G T C | 1e-26 | -6.213e+01 | 5.07% | 3.60% | 257.3bp (248.8bp) | PKNOX1/MA0782.2/Jaspar(0.697) More Information \| Similar Motifs Found | motif file (matrix) |
| 28 | C G T A A C G T A C T G A T C G A C G T G T C A A C G T A C T G A G T C C T A G | 1e-25 | -5.866e+01 | 0.24% | 0.04% | 177.0bp (126.2bp) | Oct4:Sox17(POU,Homeobox,HMG)/F9-Sox17-ChIP-Seq(GSE44553)/Homer(0.672) More Information \| Similar Motifs Found | motif file (matrix) |
| 29 | C A G T A C G T A C G T C T A G A C T G A G T C A T G C A C T G A C G T G T A C | 1e-25 | -5.804e+01 | 0.21% | 0.03% | 201.4bp (72.1bp) | NFIX/MA0671.1/Jaspar(0.734) More Information \| Similar Motifs Found | motif file (matrix) |
| 30 | A G T C A G T C A G T C C G T A C G T A A C T G A C T G A C T G A G T C A C T G A G T C A G T C | 1e-22 | -5.168e+01 | 0.19% | 0.02% | 212.9bp (133.7bp) | EBF3/MA1637.1/Jaspar(0.743) More Information \| Similar Motifs Found | motif file (matrix) |
| 31 | A C T G A G T C A G T C C G T A A G T C C G T A A G T C A C T G C G T A A C G T A G T C A C G T | 1e-22 | -5.168e+01 | 0.19% | 0.03% | 276.5bp (106.5bp) | MITF(bHLH)/MastCells-MITF-ChIP-Seq(GSE48085)/Homer(0.642) More Information \| Similar Motifs Found | motif file (matrix) |
| 32 | C G T A A C G T C G T A A C G T C G T A A G T C A G T C A G T C C G T A A C T G | 1e-22 | -5.077e+01 | 0.30% | 0.07% | 197.3bp (217.2bp) | ZNF416(Zf)/HEK293-ZNF416.GFP-ChIP-Seq(GSE58341)/Homer(0.680) More Information \| Similar Motifs Found | motif file (matrix) |
| 33 | A C T G A C T G C G T A A G C T A T C G A C T G C G T A A C G T A G T C A G T C | 1e-21 | -4.919e+01 | 0.24% | 0.04% | 241.7bp (111.3bp) | HNF6(Homeobox)/Liver-Hnf6-ChIP-Seq(ERP000394)/Homer(0.692) More Information \| Similar Motifs Found | motif file (matrix) |
| 34 | G T C A C G T A A C T G A G C T A C G T A C T G A G T C A C T G A C G T T A C G | 1e-20 | -4.706e+01 | 0.21% | 0.04% | 187.2bp (241.7bp) | Arnt:Ahr(bHLH)/MCF7-Arnt-ChIP-Seq(Lo\_et\_al.)/Homer(0.713) More Information \| Similar Motifs Found | motif file (matrix) |
| 35 | A G T C C G T A A C G T A C T G A C T G A C T G C G T A A C G T A C T G A C G T | 1e-18 | -4.156e+01 | 0.17% | 0.03% | 273.6bp (99.6bp) | PB0098.1\_Zfp410\_1/Jaspar(0.839) More Information \| Similar Motifs Found | motif file (matrix) |
| 36 | G T A C A T C G A C G T C G T A A C T G A C G T A G T C A G C T | 1e-15 | -3.636e+01 | 5.15% | 4.00% | 253.4bp (271.0bp) | Smad3(MAD)/NPC-Smad3-ChIP-Seq(GSE36673)/Homer(0.690) More Information \| Similar Motifs Found | motif file (matrix) |
| 37 | A G T C A C T G A C G T A C T G A C G T G T C A A G T C A C T G | 1e-14 | -3.435e+01 | 0.39% | 0.14% | 300.9bp (248.5bp) | EGR2/MA0472.2/Jaspar(0.679) More Information \| Similar Motifs Found | motif file (matrix) |
| 38 | A C T G A G T C A C T G A C G T A G T C A G T C A C G T A C G T C G T A A C G T A C G T C G T A | 1e-14 | -3.405e+01 | 0.21% | 0.05% | 234.9bp (154.7bp) | PH0152.1\_Pou6f1\_2/Jaspar(0.639) More Information \| Similar Motifs Found | motif file (matrix) |
| 39 | A C T G A C T G C G T A A C T G G T C A A C T G A G T C A G T C | 1e-13 | -3.173e+01 | 4.41% | 3.42% | 257.1bp (259.7bp) | ZNF341(Zf)/EBV-ZNF341-ChIP-Seq(GSE113194)/Homer(0.697) More Information \| Similar Motifs Found | motif file (matrix) |
| 40 | A C T G A C T G A C G T A C T G C G T A A C T G A C G T A C T G A C T G A C G T | 1e-13 | -3.122e+01 | 0.17% | 0.04% | 186.8bp (255.8bp) | NKX2-2/MA1645.1/Jaspar(0.769) More Information \| Similar Motifs Found | motif file (matrix) |
| 41 | A T C G A G T C A C T G C G T A C G T A A C G T C G T A A C T G | 1e-12 | -2.972e+01 | 0.41% | 0.17% | 252.3bp (150.3bp) | VENTX/MA0724.1/Jaspar(0.659) More Information \| Similar Motifs Found | motif file (matrix) |
| 42 | A G T C A C T G C T A G A G T C A C T G A G T C A C G T C G T A | 1e-12 | -2.857e+01 | 0.14% | 0.03% | 249.0bp (116.1bp) | PB0112.1\_E2F2\_2/Jaspar(0.770) More Information \| Similar Motifs Found | motif file (matrix) |
| 43 | C T G A A C T G C G T A A C G T C G T A A C G T C G T A G C T A C T G A A G T C C G T A G T C A | 1e-12 | -2.797e+01 | 0.18% | 0.04% | 182.9bp (143.8bp) | Foxf1(Forkhead)/Lung-Foxf1-ChIP-Seq(GSE77951)/Homer(0.786) More Information \| Similar Motifs Found | motif file (matrix) |
| 44 \* | C G T A A C T G C G T A A C T G C G T A A C T G C G T A A C T G | 1e0 | -2.202e+00 | 9.99% | 9.74% | 257.4bp (304.1bp) | PRDM1/MA0508.3/Jaspar(0.791) More Information \| Similar Motifs Found | motif file (matrix) |
